# Molecular Disorder in (‒)-Encecanescin

**DOI:** 10.3390/molecules19044695

**Published:** 2014-04-15

**Authors:** Benito Reyes-Trejo, Diana Guerra-Ramírez, Holber Zuleta-Prada, Rosa Santillán, María Elena Sánchez-Mendoza, Jesús Arrieta, Lino Reyes

**Affiliations:** 1 Laboratorio de Productos Naturales, Área de Química, Departamento de Preparatoria Agrícola AP 74 Oficina de Correos Chapingo, Universidad Autónoma Chapingo, Km. 38.5 Carretera México-Texcoco, Texcoco 56230, Estado de México, Mexico; E-Mails: dg_bonita33@yahoo.com.mx (D.G.-R.); hozuleta_13@hotmail.com (H.Z.-P.); 2 Departamento de Química, CINVESTAV-IPN, Apdo. Postal 14-740, México D.F. 07000, Mexico; E-Mail: rsantill@cinvestav.mx; 3 Escuela Superior de Medicina, Instituto Politécnico Nacional, Plan de San Luis y Díaz Mirón, Colonia Santo Tomás, Delegación Miguel Hidalgo, México D.F. 11340, Mexico; E-Mails: mesmendoza@hotmail.com (M.E.S.-M.); jearrval@yahoo.com.mx (J.A.); 4 Departamento de Química Orgánica, Facultad de Química, Universidad Nacional Autónoma de México, Ciudad Universitaria, Delegación Coyoacán D.F. 04510, Mexico

**Keywords:** encecanescin, DFT calculations, X-ray diffraction, FTIR and NMR spectra

## Abstract

(‒)-Encecanescin (**1**) has been isolated from the leaves of *Eupatorium aschembornianum*. Two conformers are present in the crystal structure as a result of molecular disorder. The structure of **1** was established by ^1^H- and ^13^C-NMR spectroscopy in CDCl_3_ solution using 2D NMR techniques (gHSQC, gHMBC and NOESY). A Monte Carlo random search using molecular mechanics followed by the geometry optimization of each minimum energy structure using density functional theory (DFT) calculations at the B3LYP/6–31G* level and a Boltzmann analysis of the total energies generated accurate molecular models describing the conformational behavior of **1**. The three most stable conformers **2**–**4** of compound **1** were reoptimized at the B3LYP/6-311++G(d,p) level of theory using CHCl_3_ as a solvent. Correlations between the experimental ^1^H- and ^13^C-NMR chemical shifts (δ_exp_) have been found, and the GIAO/B3LYP/6-311++G(d,p) calculated magnetic isotropic shielding tensors (σ_calc_) for conformers **2** and **3**, δ_exp_ = a + b σ_calc_, are reported. A good linear relationship between the experimental and calculated NMR data has been obtained for protons and carbon atoms.

## 1. Introduction

Chromene (2*H*-1-benzopyran) ring derivatives are often found in natural heterocycles, and some have interesting biological activities [1]. These compounds make up a new family of activators of potassium channels that are useful in the treatment of respiratory diseases as tracheal tissue relaxing agents [2]. One such benzopyran derivative isolated from *Ageratina asenii* [3] is (+)-encecanescin, a dimeric chromene with a structure similar to a previously reported compound [4]. Surprisingly, however, the authors found that the available encecanescin crystallized as a racemic mixture.

**Figure 1:**
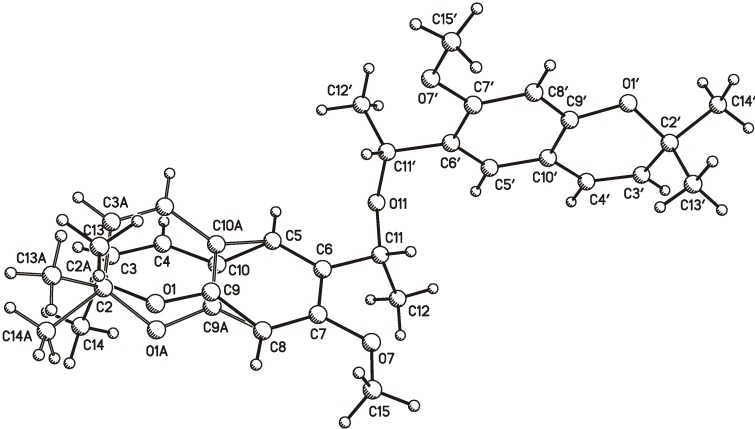
Atom numbering for (‒)-encecanescin (**1**). The segments of atoms C1, C2, C3, C4, C9, C10 and the methyl groups at C2 are disordered in the crystal and two conformers (**1a** and **1b** labeled with A) are present.

Later, crystals of (±)-encecanescin were analyzed by X-ray diffraction [5], revealing that it racemized during crystallization using 1:1 EtOAc/cyclohexane. In addition, it was possible to observe two molecules in the asymmetric unit that differ in geometry around C2. The rings attached to C2 exhibit a half-boat conformation, but differ in the orientation of the *gem*-dimethyl group present at C2. Our group recently obtained a white solid from *Eupatorium aschembornianum* by recrystallization using 95:5 hexane/EtOAc , which provided (‒)-encecanescin (**1**) for the first time as a single crystal, with the structure shown in Figure 1 [6]. However, the molecular crystal also exhibited disorder in the X-ray structure. In the present work, this disorder is explained in terms of two conformers in the solid state, and it is shown that this disorder can be deduced from quantum mechanical calculations.

## 2. Results and Discussion

(‒)-Encecanescin (**1**) was isolated using a previously reported protocol [6]. The dimeric structure of compound **1** was confirmed from the high-resolution mass spectra (MS-FAB+), which exhibited a peak at 450.2402 for C_28_H_34_O_5_. The compound was identified using NMR analysis by comparing the results with those previously reported [3,4], with the exception of the resonances for C-3, C-3'; C-4, C-4'; and C-5, C-5', which were reassigned in this study to 127.3, 122.7 and 124.0, respectively, based on gHMBC, gHSQC and NOESY spectra.

## 2.1. X-ray Crystallography

Crystals of **1** were grown from a 95:5 mixture of *n*-hexane with ethyl acetate. A crystal cut to the dimensions 0.24 × 0.20 × 0.18 mm was used for X-ray measurements at 293 K using an Enraf-Nonius KappaCCD diffractometer with graphite-monochromated λ_Mo-Ka_ = 0.71073 Å. The structure was solved by direct methods and refined by full-matrix least-square calculations based on F^2^. Crystallographic calculations were performed using SHELXL-97 [7]. The details of the crystal structure determinations and refinements are presented in Table 1. The best results were obtained for the disordered model in the crystal, and two conformers (**1a**, **1b**) are found (Figure 1). The rings containing C2 in **1a** and **1b** show a half-boat conformation but differ in the orientation of the flag atom C2.

**Table 1:** Crystal data and structure refinement for (‒)-encecanescin (**1**).

Empirical formula	C_28_H_34_O_5_
Formula weight	450.2402
Temperature	293(2) K
Wavelength	0.71073 Å
Crystal system, space group	monoclinic, P2_1_/c
Unit cell dimensions	a = 11.0930(2) Å alpha = 90°
	b = 8.4352(2) Å beta = 94.622(11)°
	c = 27.5559(11) Å gamma = 90°
Volume	2570.07(14) Å^3^
ZCalculated density	40.851 Mg/m^3^
Absorption coefficient	0.061 mm^−1^
F(000)	724
Crystal size	0.24 × 0.20 × 0.18 mm
Theta range for data collection	2.46° to 27.49°
Limiting indices	−14 ≤ h ≤ 13, −10 ≤ k ≤ 10, −35 ≤ l ≤ 28
Reflections collected/ unique	14836/5814 [R(int) = 0.0892]
Completeness to theta = 27.49	98.7%
Refinement method	Full-matrix least-squares on F^2^
Data/restraints/parameters	5814/216/379
Goodness-of-fit on F^2^	0.918
Final R indices	[I > 2sigma(I)] R1 = 0.0671, wR2 = 0.1293
R indices (all data)	R1 = 0.2389, wR2 = 0.1772
Largest diff. peak and hole	0.160 and −0.166 e.A^−3^

## 2.2. B3LYP Calculations

**Figure 2:**
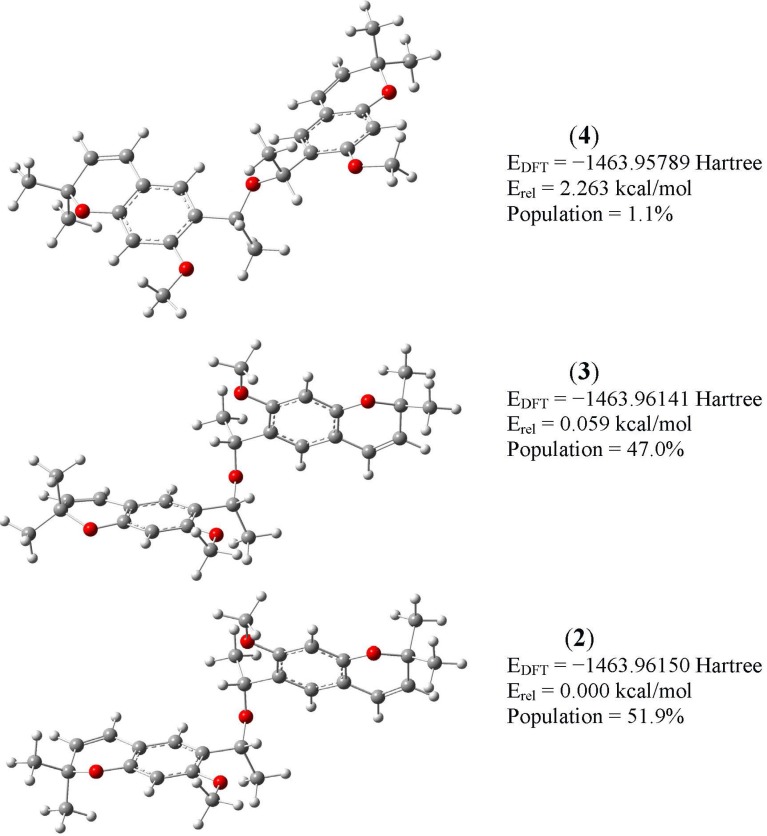
Conformational distribution of (‒)-encecanescin (**1**).

The theoretical conformational distribution of **1** was obtained by a Monte Carlo random search. A total of 10 minimum energy structures were found within a molecular mechanics energy range of 10 kcal·mol^−1^. All of these structures were subjected to geometry and energy optimization by density functional theory (DFT) calculations employing the B3LYP/6–31G* basis set. According to these calculations, the original group of 10 structures was reduced to a group of three (within a 0–3 kcal·mol^−1^ range), as seven conformers appeared as duplicates. These three structures were submitted to geometry reoptimization using DFT calculations at the B3LYP/6-311++G(d,p) level of theory in a CHCl_3_ solution. Figure 2 shows the total DFT energy in solution, the relative energy and the conformational population of the three optimized conformers of **1** (**2**, **3** and **4**), which account for 99.99% of the conformational population according to the DFT total energy values. Geometry optimizations included a frequency calculation to verify that an energy minimum had been reached. Given that conformer **4** has a relative energy of 2.263 kcal·mol^−1^, its contribution to the equilibrium (*ca.* 1.1%) can be neglected.

The selected calculated B3LYP bond lengths, bond angles and torsion angles are given in Table 2. Most of the calculated bonds are slightly longer than the experimental ones, except C(10)-C(5), C(10)-C(9), C(2)-O(1) and C(9)-C(8), which are shorter. The calculated bond angles agree with the experimental values within 1.3°, excluding angles within the rings containing C2, ranging from 5.2° to 35.6°. The largest differences between the X-ray and B3LYP data are in the torsion angles, which vary from 2.5° to 69.6°. The B3LYP calculations accurately reproduce the signs of the torsion angles.

**Table 2:** Selected bond lengths (Å), bond angles and torsion angles (°) for (−)-encecanescin (**1**) determined by X-ray diffraction and B3LYP calculations at the 6-311++G(d,p) level of theory.

Parameter	1	2	3
*Bond length*			
C(10)-C(5)	1.401(15)	1.40026	1.4005
C(10)-C(4)	1.436(15)	1.45559	1.4568
C(10)-C(9)	1.482(16)	1.40225	1.4032
C(4)-C(3)	1.310(10)	1.33793	1.3386
C(3)-C(2)	1.38(2)	1.51363	1.5129
C(2)-C(13)	1.446(15)	1.52796	1.53535
C(2)-C(14)	1.451(16)	1.53726	1.52713
C(2)-O(1)	1.486(13)	1.46334	1.46937
O(1)-C(9)	1.36(2)	1.36414	1.36453
C(9)-C(8)	1.44(2)	1.39292	1.39302
C(5)-C(6)	1.377(4)	1.38931	1.39071
C(6)-C(7)	1.396(4)	1.40947	1.41067
C(3')-C(2')	1.494(4)	1.51363	1.51290
C(2')-C(13')	1.521(4)	1.52796	1.53535
C(2')-C(14')	1.521(4)	1.53726	1.52713
*Bond angle*			
C(5)-C(10)-C(4)	126.7(9)	124.31413	124.28231
C(5)-C(10)-C(9)	113.3(13)	118.07731	118.01906
C(4)-C(10)-C(9)	112.7(14)	117.54869	117.66232
C(3)-C(4)-C(10)	122.6(8)	120.34825	120.33249
C(4)-C(3)-C(2)	125.4(10)	121.02151	121.32649
C(3)-C(2)-C(13)	123.9(16)	111.64217	110.71254
C(3)-C(2)-C(14)	76.0(9)	110.62884	111.60611
C(13)-C(2)-C(14)	157.0(19)	111.16920	111.19836
C(3)-C(2)-O(1)	115.7(14)	110.60774	110.50808
C(13)-C(2)-O(1)	75.0(8)	104.5417	108.01913
C(14)-C(2)-O(1)	108.6(10)	108.03583	104.57564
C(9)-O(1)-C(2)	118.6(13)	118.74609	119.00702
O(1)-C(9)-C(8)	119.7(11)	117.56225	117.58616
O(1)-C(9)-C(10)	123.7(17)	121.35132	121.26106
C(3')-C(2')-C(13')	111.4(3)	110.60783	110.71254
C(3')-C(2')-C(14')	111.2(3)	111.56751	111.60611
C(13')-C(2')-C(14')	111.0(3)	111.18802	111.19836
C(13)-C(2)-O(1)	106.7(3)	107.99372	108.01913
*Dihedral angle*			
C(4)-C(3)-C(2)-C(13)	80.0(17)	140.83879	93.83749
C(4)-C(3)-C(2)-C(14)	−112.9(12)	−94.753	−141.72175
C(13)-C(2)-O(1)-C(9)	−115.3(12)	−156.22747	−84.38296
C(14)-C(2)-O(1)-C(9)	88.5(17)	85.28967	157.09634
C(13')-C(2')-C(3')-C(4')	97.5(3)	94.55224	93.83749
C(14')-C(2')-C(3')-C(4')	−138.1(3)	−141.02837	−141.72175
C(13')-C(2')-O(1')-C(9')	−88.3(3)	−85.26823	−84.38296
C(14')-C(2')-O(1')-C(9')	153.8(2)	156.2661	157.09634

## 2.3. FTIR and Raman Spectra

The observed and calculated harmonic frequencies of the two conformers (**2** and **3**) of (‒)-encecanescin (**1**) and their tentative assignments are presented in Table 3. A comparison of the calculated and experimental frequencies reveals important differences. Two factors may be responsible for the disagreements between the experimental and computed spectra of the studied structures. The first is that the experimental spectrum was recorded for the molecule in the solid state, while the computed spectra correspond to isolated molecules in CHCl_3_ solution. The second is the fact that the experimental values correspond to anharmonic vibrations, while the calculated values correspond to harmonic vibrations. The overestimation of the computed wavenumbers is quite systematic, and a scaling procedure was used to obtain the predicted frequencies [8,9].

**Table 3:** Experimental and calculated (B3LYP/6-311G(d,p)) vibrational frequencies of (−)-encecanescin (**1**).

Raman	IR_exp_	IR_calc_	INT	IR_calc_	INT	Proposed assignment
	1	2		3		
3043.66	3041 w	3177	15.7	3176	10.4	υC-H Ar
2983.39	2974 m	3131	17.8	3131	35.9	υ_as_ CH_3_ methoxy
2938.11	2932 w	3106	24.7	3106	23.8	υ_as_ CH_3_ *gem*
1645.95	1644 vw	1651	298.0	1651	3.2	υ_s_ HC=CH ring
1621.54	1615 m	1599	31.4	1600	26	υ_s_ HC=CH ring
1578.38	1576 s	1523	155.4	1524	181.6	βC-H Ar+CH_3_
1499.45	1492 w	1493	18.7	1493	23.3	γCH_3_ methoxy
	1462 vw	1479	1.8	1479	2.4	ωCH_3_ methoxy
1432.21	1443 vw	1457	2.4	1456	1.1	βring, Ar-O-R
	1380 m	1382	52.6	1383	49.3	ρHC=CH ring/ωAr-C-H-OCH_3_
1362.52	1360 m	1372	12.6	1374	26.1	γC-H
1307.86	1303 s	1303	154.2	1303	159.1	υ_as_ HC=CH Ar
	1279 m	1284	35.4	1285	27.8	βAr, ring
1239.18	1230 m	1231	33.3	1232	40.8	βHC=CH ring/υ_as_ CH_3_ *gem*
	1196 s	1214	158.7	1214	167.4	ρCH_3_ methoxy, CH_3_ *gem*
1173.11	1163 m	1178	58.3	1180	89.3	βHC=CH ring, C-H Ar
1114.62	1123 vs	1145	625.3	1145	615.6	υ_as_C-O-C
	1093 m	1104	100.6	1099	37.7	τCH_3_-C-O-C-CH_3_
	1074 vs	1087	218.5	1087	224.4	ρCH_3_/υ_as_ C-O-C
	1029 m	1048	39.8	1048	44.3	υCH_3_-CH,CH_3_-O
	1013 s	1033	88.9	1033	77.6	υ_as_Ar-O-CH_3_/τHC-CH_3_
950.96	959 m	967	28.5	967	43.7	υ_s_HC-C(CH_3_)_2_-O, CH_3_-CH-O
884.95	894 m	902	55.6	902	55.5	τCH_3_ *gem*
804.36	801 vw	809	3.1	809	1.1	τAr-O-R, ring

The abbreviations used are: vs, very strong; s, strong; m, medium; w, weak; vw, very weak; υ, stretching (s, symmetric; as, asymmetric); β, in-plane bending; γ, out-of-plane bending; ω, wagging; ρ, rocking; τ, twisting.

## 2.4. ^1^H- and ^13^C-NMR Spectra

**Figure 3:**
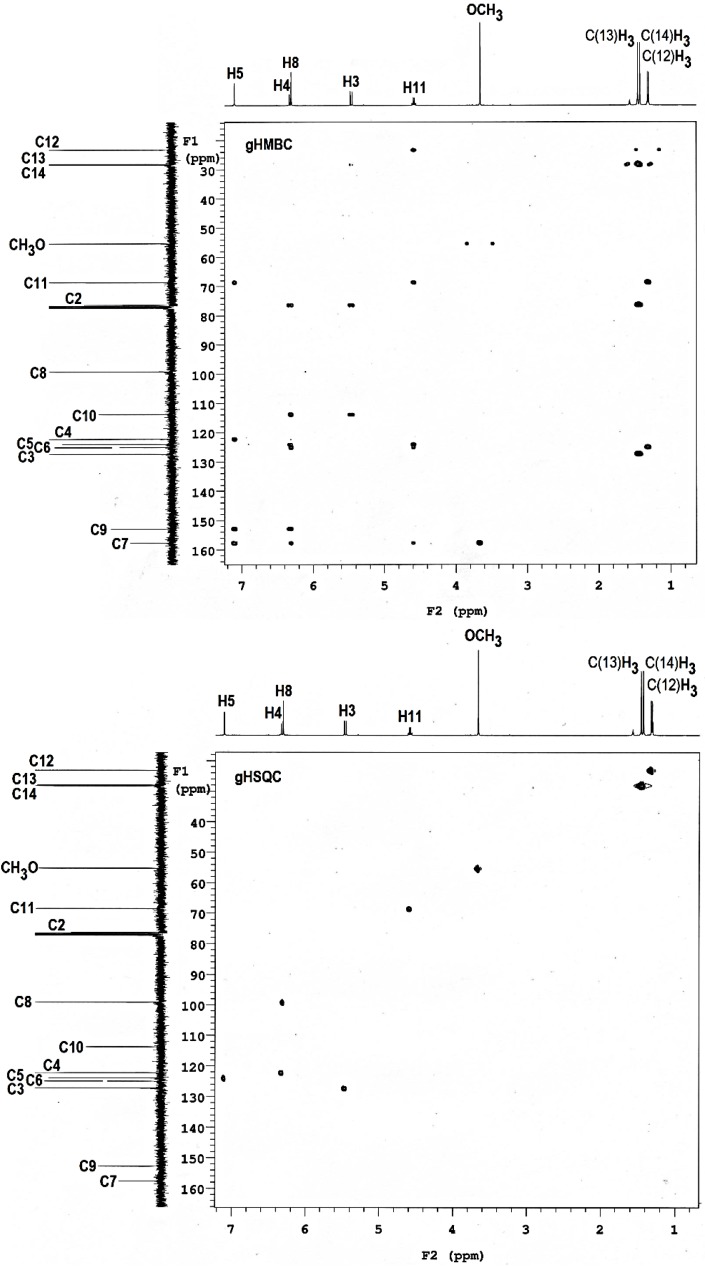
gHMBC and gHSQC spectra of (‒)-encecanescin (**1**) in CDCl_3_.

The ^1^H- and ^13^C-NMR signals of **1** were assigned based on the observed gHSQC, gHMBC and NOESY correlations in CDCl_3_ [10] and are listed in Table 4 and Table 5. The gHSQC spectrum (Figure 3) shows cross peaks between the resonances of ^1^H and those of the ^13^C atoms to which the protons are attached. The horizontal axis corresponds to the ^1^H spectrum and the vertical one to the ^13^C spectrum. On the other hand, in the HMBC spectrum, correlations between the protons or carbons through two and three bonds are observed (Figure 3).

**Table 4:** Carbon-13 chemical shifts (δ, ppm) in CDCl_3_ and calculated GIAO nuclear magnetic shielding (σ_cal_) for (−)-encecanescin (**1**). The predicted GIAO chemical shifts were computed from the linear equation δ_exp_ = a + b·σ_calc_ with a and b determined from the fit to the experimental data.

Atom	δ_exp_	δ_pred _(2)	δ_pred_ (3)	σ_calc_ (2)	σ_calc_ (3)
C(2)	78.3	80.7	80.2	98.47	98.84
C(3)	127.3	124.1	124.5	52.53	52.00
C(4)	122.7	124.9	124.3	51.68	52.19
C(5)	124.0	124.4	123.9	52.22	52.58
C(6)	125.1	126.3	125.7	50.27	50.70
C(7)	157.7	158.8	157.6	15.85	17.03
C(8)	99.2	96.2	95.7	82.07	82.46
C(9)	152.8	153.6	154.5	21.32	20.25
C(10)	113.8	113.4	114.0	63.91	63.12
C(11)	68.6	68.5	69.3	111.29	110.30
C(12)	23.2	24.7	24.5	157.67	157.66
C(13)	28.3	29.5	27.0	152.57	155.04
C(14)	28.1	26.8	29.5	155.41	152.43
C(15)	55.3	53.8	53.7	126.87	126.85
C(2')	78.3	80.7	80.2	98.47	98.84
C(3')	127.3	124.5	124.5	52.16	52.00
C(4')	122.7	125.0	124.3	51.64	52.19
C(5')	124.0	123.7	123.9	53.01	52.58
C(6')	125.1	125.0	125.7	51.61	50.70
C(7')	157.7	157.8	157.6	16.96	17.03
C(8')	99.2	95.7	95.7	82.58	82.46
C(9')	152.8	153.3	154.5	21.67	20.25
C(10')	113.8	113.9	114.0	63.28	63.12
C(11')	68.6	68.8	69.3	110.98	110.30
C(12')	23.2	24.4	24.5	157.92	157.66
C(13')	28.3	27.0	27.0	155.15	155.04
C(14')	28.1	29.6	29.5	152.41	152.43
C(15')	55.3	53.7	53.7	126.98	126.85
A		173.81	173.68		
B		−0.946	−0.946		
r^2^		0.9986	0.9986		

Starting from the characteristic resonance of the H-15 methoxyl proton (δ 3.67), it was possible to assign the resonance of the sp^2^ carbon C-7 (δ 157.7) based on its gHMBC correlation with H-15. On the other hand, the signal at δ 6.32 was assigned to H-8 due to its cross peak with CH_3_O (δ 3.67) in the NOESY plot. Analogously, the methine proton resonating at δ 4.59 produces cross peaks with carbons C-5 (δ 124.0) and C-6 (δ 125.1), thus revealing its position on C-11. The resonance of methyl protons C(12)H_3_ at δ 1.46 is coupled through two bonds to the carbon C-11. Furthermore, C-7 correlates in the gHMBC through two and three bonds with the protons at δ 6.32 and δ 7.10; therefore, these signals must be assigned to the protons H-8 and H-5, respectively. Similarly, relevant cross peaks were observed for proton H-5 at δ 7.10 through three bonds with the carbons C-4 at δ 122.7 and C-11 at δ 68.6, confirming its assignment to H-5. In addition, the signal at δ 152.8 correlates in the gHMBC through three bonds with the protons H-5 at δ 7.10 and H-4 at δ 6.34; therefore, this signal must be assigned to carbon C-9. The resonances of the methyl protons C(13)H_3_ and C(14)H_3_ at δ 1.46 and δ 1.43 are coupled through three bonds to carbon C-3 at δ 127.3. Thus, the signal at δ 5.46 was assigned to H-3 based on its cross peak with C(13)H_3_ and C(14)H_3_ and H-4 at δ 6.34 in the NOESY plot. The signal at δ 113.8 correlates in the gHMBC through three bonds to the carbon with the protons H-8 and H-3; therefore, this signal must be assigned to carbon C-10. Accordingly, the remaining carbons C-8 and C-2, resonating at δ 99.2 and δ 78.3, respectively, were assigned based on the gHSQC correlations.

**Table 5:** Experimental chemical shifts (δ_exp_, CDCl_3_) *vs.* the isotropic magnetic shielding tensors (σ_calc_) from the GIAO/B3LYP/6-311++G(d,p) calculations for encecanescin (**1**); δ_exp_ = a+b·σ_calc_: (**a**) ^13^C (a = 173.81; b = −0.946; r^2^ = 0.9986) and (**b**) ^1^H (a = 32.101; b = −1.0124; r^2^ = 0.9952).

Atom	δ_exp_	δ_pred_ (2)	δ_pred_ (3)	σ_calc_ (2)	σ_calc_ (3)
H(3)	5.46	5.44	5.44	26.34	26.34
H(4)	6.34	6.25	6.39	25.54	25.33
H(5)	7.10	7.34	7.21	24.46	24.46
H(8)	6.32	6.15	6.05	25.64	25.69
H(11)	4.59	4.68	4.69	27.08	27.15
H(12)	1.31	1.26	1.29	30.46	30.78
H(13)	1.46	1.69	1.29	30.04	30.77
H(14)	1.43	1.41	1.64	30.32	30.39
H(15)	3.67	3.48	3.68	28.28	28.22
A		32.076	30.141		
B		−1.0114	−0.9376		
r^2^		0.9956	0.9956		

**Figure 4:**
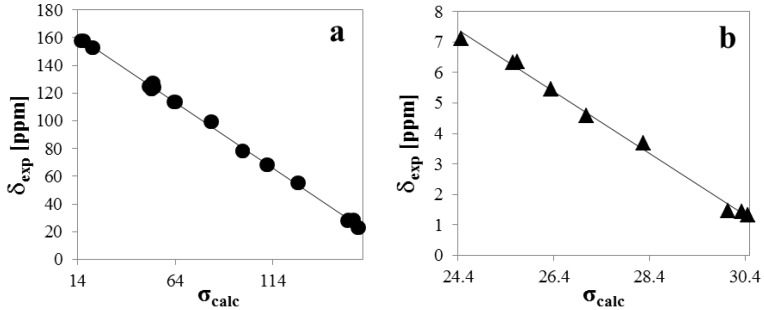
Experimental chemical shifts (δ_exp_, CHCl_3_) *vs.* the isotropic magnetic shielding tensors (σ_calc_, weighed by taking into account the Boltzmann distribution) from the GIAO/B3LYP/6-311++G(d,p) calculations for encecanescin (1); δ_exp_ = a + b·σ_calc_: (**a**) ^13^C (a = 173.70; b = −0.956; r^2^ = 0.998) and (**b**) ^1^H (a = 31.16; b = −0.987; r^2^ = 0.997).

The relationship between the experimental ^13^C and ^1^H chemical shifts (δ_exp_) and the GIAO (gauge-independent atomic orbitals) magnetic isotropic shielding constants (σ_calc_) calculated for conformers **2** and **3** in CHCl_3_ are generally linear and are well described by the equation δ_exp_ = a + b·σ_calc_ [11]. The slope and intercept of the least-squares correlation line (Figure 4a,b, Table 4 and Table 5) are utilized to scale the GIAO magnetic isotropic shielding constants, σ_calc_, and to predict the chemical shifts, δ_pred_ = a + b·σ_calc_. The correlations between the experimental chemical shifts and calculated magnetic isotropic shielding constants are generally better for carbon-13 atoms than for protons; however, in this case, the correlations are good for both carbons and protons. This finding can be explained by the absence of hydrogen bonds and other strong interactions that mainly affect outer H atoms. The magnetic isotropic shielding constants confirm the correct assignments of the chemical shifts to the aforementioned atoms.

## 3. Experimental

## 3.1. General

The infrared spectrum was recorded on a Varian FT-IR spectrometer (Palo Alto city, CA, USA). The Raman spectrum of a crystalline sample was measured using a Thermo Scientific DXR Raman microscope (Madison, WI, USA) equipped with a 532 nm laser with a power of 10 mW and an exposure time of 104 s. High-resolution mass spectroscopy was performed using a JEOL spectrometer (model 102 ASX, Jeol).

Diffraction data were measured using an Enraf-Nonius KappaCCD diffractometer (Nonius, Delft, The Netherlands) with graphite-monochromated λ_Mo-Kα_ = 0.71073 Å. Frames were collected at T = 293 K ω/φ rotation. The direct methods SHELXS-86 and SIR-2004 were used to solve the structure, and the SHELXL-97 program package was used for refinement and data output. CCDC 996389 for (−)-encecanescin (**1**) contains the supplementary crystallographic data for this paper. These data can be obtained free of charge from http://www.ccdc.cam.ac.uk/conts/retrieving.html (or from the Cambridge Crystallographic Data Centre, 12, Union Road, Cambridge CB2 1EZ, UK; [deposit_reply@ccdc.cam.ac.uk]).

The ^13^C- and ^1^H-NMR spectra were recorded on an Agilent 400 MR DD2 spectrometer (Santa Clara, CA, USA) operating at 100 MHz for ^13^C and 400 MHz for ^1^H. The ^13^C and ^1^H chemical shifts were measured in CDCl_3_ relative to TMS as an internal standard. Typical conditions for the proton spectra were as follows: pulse width of 45°, acquisition time of 2.5 s, FT size of 32 K and digital resolution of 0.3 Hz per point. Typical conditions for the carbon spectra were as follows: pulse width of 45°, FT size of 65 K and digital resolution of 0.5 Hz per point. The number of scans varied from 1200 to 10,000 per spectrum. All proton and carbon-13 resonances were assigned by ^1^H (NOESY) and ^13^C (gHSQC, gHMBC), respectively. All 2D NMR spectra were recorded at 298 K on the Agilent 400 MR DD2 spectrometer operating at 100 MHz (^13^C) and 400 MHz (^1^H), with a FT size of 2 × 2 K and a digital resolution of 0.3 Hz per point.

## 3.2. Materials

*Eupatorium aschembornianum* leaves were collected in San Juan Tlacotenco, Tepoztlan, Morelos State, México, during August 2007. A specimen from the original collection can be found in “Jorge Espinosa Herbarium-Hortorio” in the Biology Area of Chapingo Autonomous University, with voucher number 1835.

## 3.3. Methods

Hexane extract (40 g) from the leaves of *E. aschembornianum* were chromatographed over silica gel (250 g) with increasing solvent polarity, starting with hexane and increasing the polarity with ethyl acetate. Fractions 17-40 eluted with hexane/EtOAc (95:5) provided a white solid (2.3 g, mp = 148−150 °C) identified as (‒)-encecanescin (**1**), [α]_D_ 25° (CHCl_3_, c2.21 g/100 mL): 589 (−0.4), 578 (−0.4), 546 (−0.5), 436 (−0.8), 365 (−1.2). MS-FAB+: observed 450.2402, calculated 450.2406 for C_28_H_34_O_5_. IR (CHCl_3_): υmax = 3010, 1640, 1385, 1140 cm^−1^. ^1^H-NMR (CDCl_3_): δ = 7.10 (s, H-5, H-5'), 6.34 (d, *J* = 9.3 Hz, H-4, H-4'), 6.32 (s, H-8, H-8'), 5.46 (d, *J* = 9.3 Hz, H-3, H-3'), 4.59 (q, *J* = 6.3 Hz, H-11, H-11'), 3.67 (s, H-15, H-15'), 1.46 (s, H-13, H-13'), 1.43 (s, H-14, H-14'), 1.31 (d, *J* = 6.3 Hz, H-12, H-12'). The ^13^C-NMR data (Table 4) correspond to those published for encecanescin [3], with the exception of the resonances for C-3, C-3'; C-4, C-4'; and C-5, C-5', which were reassigned in this study to 127.3, 122.7 and 124.0, respectively.

## 3.4. Computational Calculations

The conformational search for **1** was carried out using the Monte Carlo protocol [12] with the MMFF94 force field as implemented in the Spartan 08 program (Wavefunction, Inc., Irvine, CA, USA). The DFT calculations at the B3LYP/6-31G(d) level of theory [13,14], followed by reoptimization at the B3LYP/6-311++G(d,p) [15] level using the SMD solvent model [16], were performed using the Gaussian 09 package [17]. The NMR isotropic magnetic shielding tensors were calculated using the standard gauge-independent atomic orbital (GIAO) approach [11,18] in Gaussian 09.

## 4. Conclusions

(−)-Encecanescin (**1**) has been isolated from leaves of *Eupatorium aschembornianum*. The structure of **1** was established by X-ray diffraction and characterized by FTIR, Raman and NMR spectroscopy and DFT calculations. The X-ray analysis showed that the molecule is non-planar and is present as a mixture of two conformers in the crystal (**2** and **3**). Molecular modeling of **1** using the Monte Carlo protocol followed by geometry optimization at the B3LYP 6-31G(d,p) level of theory and a Boltzmann analysis of the total energies confirmed that **2** and **3** are the two most stable conformers of **1**. Good correlations between the experimental ^1^H and ^13^C chemical shifts in CHCl_3_ and the GIAO/B3LYP/6-311++G(d,p) calculated magnetic isotropic shielding tensors for both conformers (δexp = a + b·σ_calc_) confirmed the geometry of **1**.

## Supplementary Materials

Copies of the room-temperature solid-state FTIR spectra, Raman spectra and calculated vibrational spectra in CHCl_3_ solution of two conformers (**2** and **3**) of (−)-encecanescin (**1**) and a magnification of the gHSQC spectrum of (−)**-**(**1**) at 400 MHz in CDCl_3_ are available. Supplementary materials can be accessed at: http://www.mdpi.com/1420-3049/19/4/4695/s1.
