# Supplementary material for: Molecular Disorder in (‒)-Encecanescin
Source: Molecules. 2014 Apr 15;19(4):4695–707. doi: 10.3390/molecules19044695 (PMC6271977; doi:10.3390/molecules19044695)
Supplement: Supplementary file 1 [file molecules-19-04695-s001.pdf]

Supplementary Materials

Figure S1. (−)-Encecanescin (**1**): (**a**) Raman, (**b**) FTIR and (**c**) calculated spectra.

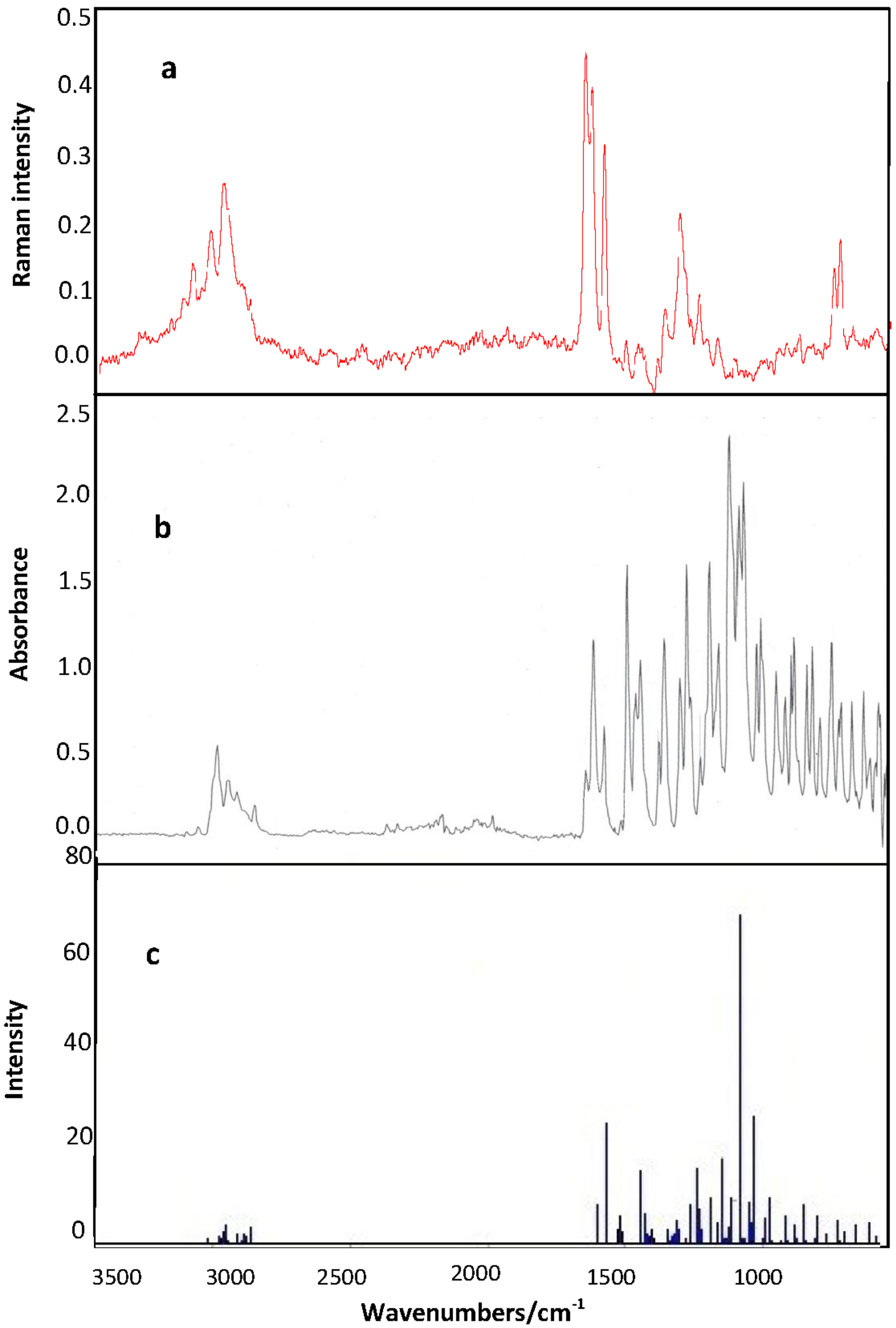

Figure S2. Magnification of the gHSQC spectrum of (−)-encecanescin (**1**) at 400 MHz in $CDCl_3$.

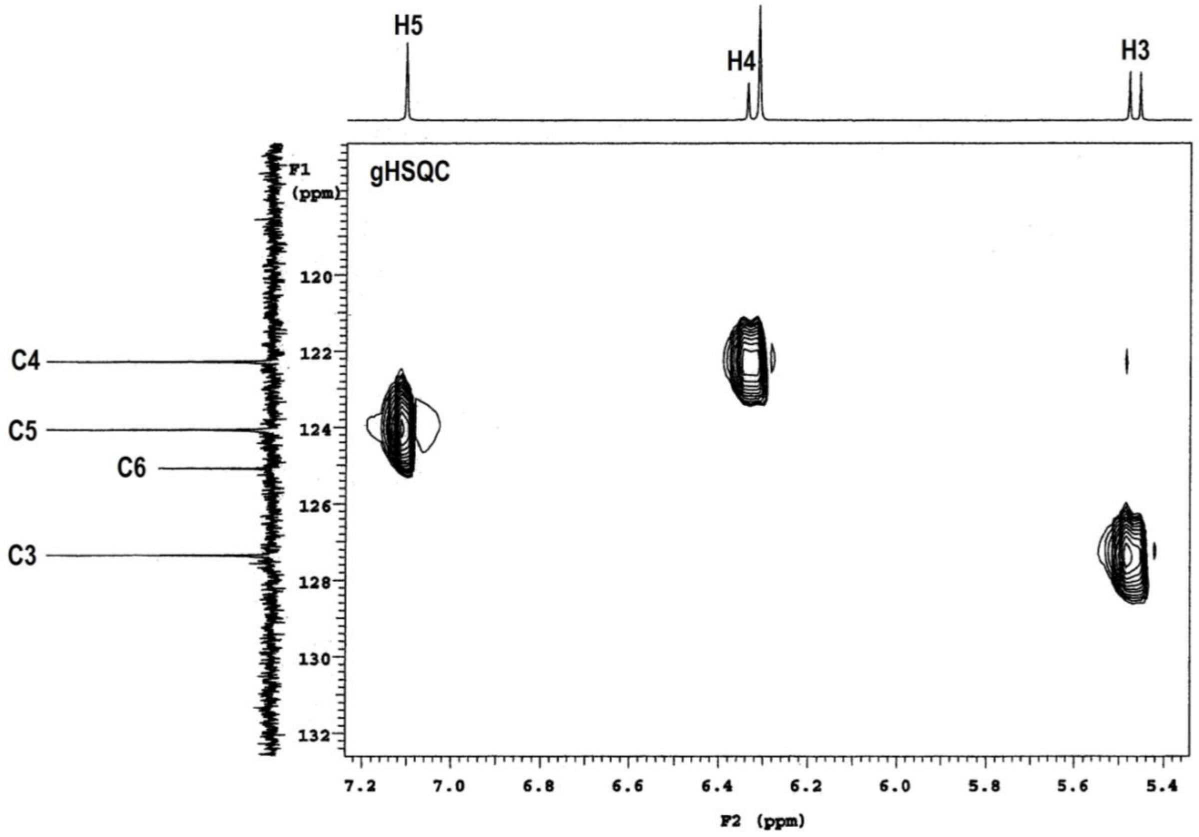

© 2014 by the authors; licensee MDPI, Basel, Switzerland. This article is an open access article distributed under the terms and conditions of the Creative Commons Attribution license (http://creativecommons.or g/licenses/by/3.0/).
